# Understanding sand fly sampling methods: sticky traps are attraction-based and not interceptive sampling tools of *Phlebotomus orientalis*

**DOI:** 10.1186/s13071-020-04249-1

**Published:** 2020-07-31

**Authors:** Dia-Eldin Elnaiem, Altayeb Khogali, Bashir Alsharif, Osman Dakein, Tayseer Jibreel, Mohamed Hassan, Hassan H. Edries, Hanan Elhadi, Bakri Elnur, Omran F. Osman, Margriet den Boer, Jorge Alvar, Noteila M. Khalid

**Affiliations:** 1 Department of Natural Sciences, University of Maryland Eastern Shore, Princess Anne, MD 21853 USA; 2 Blue Nile Health Institute for Communicable Diseases, Gezira University, Wad Medani, Sudan; 3 Medical Entomology Department, Federal Ministry of Health, Khartoum, Sudan; 4 Department of Zoology, Faculty of Science, University of Khartoum, Khartoum, Sudan; 5 Kalar azar Research Centre, Faculty of Medicine and Health Sciences, University of Gedarif, Gedarif, Sudan; 6 Ministry of Health, Gedarif state, Gedarif, Sudan; 7 KalaCORE, London, UK; 8 Drugs for Neglected Diseases Initiative, Geneva, Switzerland; 9 Department of Zoology, Ibn Sina University, Khartoum, Sudan

**Keywords:** Sand flies, VL, Surveillance, East Africa, *Phlebotomus orientalis*, Sticky traps

## Abstract

**Background:**

Sticky traps are generally viewed as interceptive sand fly sampling methods; although no previous experimental evidence has supported this assumption. In this study, we tested this assumption experimentally for *Phlebotomus orientalis*, the principal vector of visceral leishmaniasis in East Africa, and propose an explanation for the highly male-biased collection of sticky traps.

**Methods:**

A number of field experiments were carried out in March–June 2016–2019, in Gedarif state, eastern Sudan. In the first experiment, we compared numbers of *P. orientalis* caught on sticky traps made of black, red, transparent, white, yellow, green and blue A4 size papers set simultaneously at different lunar light conditions. In the second and third experiments, we compared numbers of *P. orientalis* captured on sticky traps placed side-by-side horizontally or vertically on the ground, or horizontally on a 15 cm height stool. We also witnessed mating behaviour of sand flies following their landing on un-sticky papers placed on the ground.

**Results:**

*Phlebotomus orientalis* showed significant attraction to white, yellow and transparent traps, with negligible numbers caught on the black and the red traps. Similarly, significantly higher numbers of *P. orientalis* were attracted to the horizontal traps, resulting in an 8-fold increase in sand fly trapping efficacy as compared to the vertical traps. Placing the traps on the stools resulted in significant reduction in this attraction. In contrast to the sticky traps that captured only very few females; we found that when male sand flies land on un-sticky white paper they successfully lure females and copulate with them.

**Conclusions:**

We demonstrate that, for *P. orientalis*, sticky traps are more attractant-based than interception-based sampling tools. Further, our findings support the notion that males of this sand fly species likely utilize the bright surface of the trap papers to perform mating rituals that attract the females for copulation. However, pre-mature death in the sticky oil hampers the completion of these rituals, and thus results in failure to attract the females. These findings inform our understanding of *P. orientalis* behaviour and have important implications for optimization of sticky trap design for vector surveillance purposes.
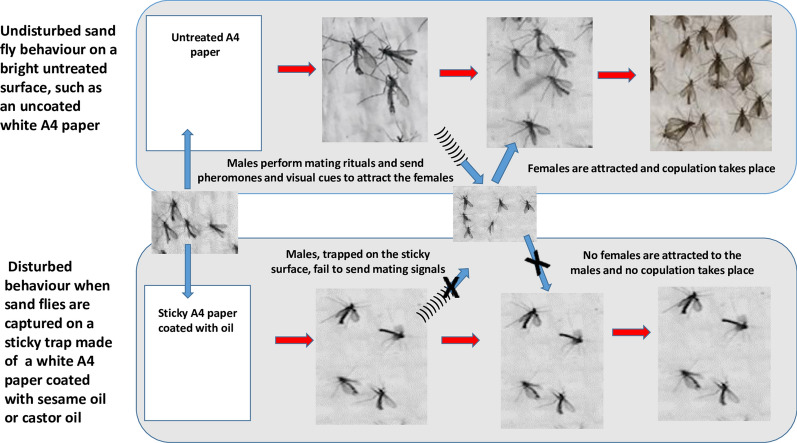

## Background

Sand flies (class: Insecta; order: Diptera, family: Psychodidae) are important haematophagus insects that transmit the pathogens responsible for leishmaniasis, bartonellosis, sand fly fever and vesicular stomatitis [1, 2]. The most important of these diseases is leishmaniasis, a multi-spectrum neglected tropical disease that manifests as long lasting cutaneous ulcers (cutaneous leishmaniasis, CL), mucocutaneous lesions (mucocutaneous leishmaniasis, MCL), or visceral infection (visceral leishmaniasis, VL). Leishmaniasis has a wide global distribution with over one billion people at risk of infection in 98 countries [3]. VL, caused by members of the *L. donovani* complex, is considered to be the most serious form of leishmaniasis. In absence of timely diagnosis and treatment, VL has a fatal outcome. There are an estimated 50,000–200,000 cases of VL annually with 5–10% mortality. Visceral leishmaniasis has a wide distribution in Latin America, Africa, Europe and Asia, with 90% of cases occurring in India, Sudan, South Sudan Brazil, Ethiopia, Kenya and Somalia [4].

*Phlebotomus orientalis* is the principal vector of VL in East Africa [5]. This sylvatic sand fly species is abundant in woodland habitat dominated by *Balanites aegyptiaca* and/or *Acacia seyal* trees that grow on black cotton soil. In villages, the vector rarely enters inside huts/rooms but can be quite abundant in household courtyards (outdoor site) or peri-domestic habitats [6, 7]. In Sudan, Ethiopia, and the Republic of South Sudan, *P. orientalis* shows marked seasonality, reaching peak abundance at late dry season (March-June) and disappears during months of heavy rains (August-September) [6–9].

Difficulties in finding sand fly breeding sites have precluded studies on their immature stages under natural conditions. Thus, sampling of adult populations and studies of laboratory colonies provide most of the current information on the ecology and biology of sand flies [10].

Adult sand flies are small nocturnal insects with a weak flight pattern; best described as short hops close to ground level [11]. Whereas both sexes feed on sugar from plant origin, females require a meal of vertebrate blood for egg nourishment and maturation. During the day, adults rest in humid places that vary by species and ranges from inside human dwellings to crevices in trees and cracks in the soil [10]. Mating takes place near or on the animal host, and in other lekking sites [12].

Efficacious, reliable and inexpensive traps are indispensable for monitoring sand fly populations, understanding their biology and ecology, and evaluating control operations targeting vectors of leishmaniasis. Such traps have been classified under two main categories: interceptive traps, which capture adults randomly by blocking their flight path; and attractant-based traps, which lure sand flies by different types of light, animal baits or other attractants (e.g. CO_2_ or kairomones) [12]. While CDC light traps are used as attractant-based sand fly sampling devises, sticky paper traps have been considered to operate as interceptive tools [12, 13].

Sand fly sticky traps are made of un-waxed cards or paper/metal sheets, coated with castor oil or other viscous oils, and are placed vertically in the flight path of sand flies [14, 15]. In addition to the advantages of being inexpensive and easy to set up, no electrical power supply is needed to operate these traps. Therefore, unlike the CDC traps, they hardly fail during operation. However, sticky traps are limited by male-biased collection, which has been observed with a number of species, including *P. orientalis* [6, 7, 9, 16, 17].

Although it is widely accepted that the sand fly sticky trap is an interceptive sampling method, this classification is not corroborated by experimental evidence or prior literature and yet has important implications for vector surveillance. Most notably, field workers place these traps on vertical axes with the intention of intercepting the flies on their presumed flight path. As we will see from the results of this present study, the vertical placement likely results in significant underestimation of *P. orientalis* vector abundance.

In this study we used a systematic experimental approach to test the notion that sticky-paper traps are interceptive sampling devices and address the intriguing question of their male-biased collection. First, we used coloured sticky paper traps to see whether sand flies are attracted to specific colour(s) or randomly trapped on any of the sticky papers. Second, we compared trapping efficacy on sticky papers that were placed side by side horizontally or vertically on the ground. We tested the hypothesis that if sticky traps (STs) were interceptive, there would be higher numbers of sand flies on the vertical traps than on the horizontal traps. We also assessed the effect of height from the ground on the trapping efficacy of horizontal traps. Finally, by visual observation, we tested two additional hypotheses: firstly, male *P. orientalis* use the bright sticky traps as a mating arena; and secondly, the reduced number of females in sticky traps is due to the premature death of males, and their failure to complete successful courtship behaviour that lures females. The findings of this study have important implications, not only for optimization of the design of the sticky traps, but also for understanding the behaviour of *P. orientalis* and planning suitable control measures of this important vector of VL.

## Methods

### Study area

**Fig. 1:**
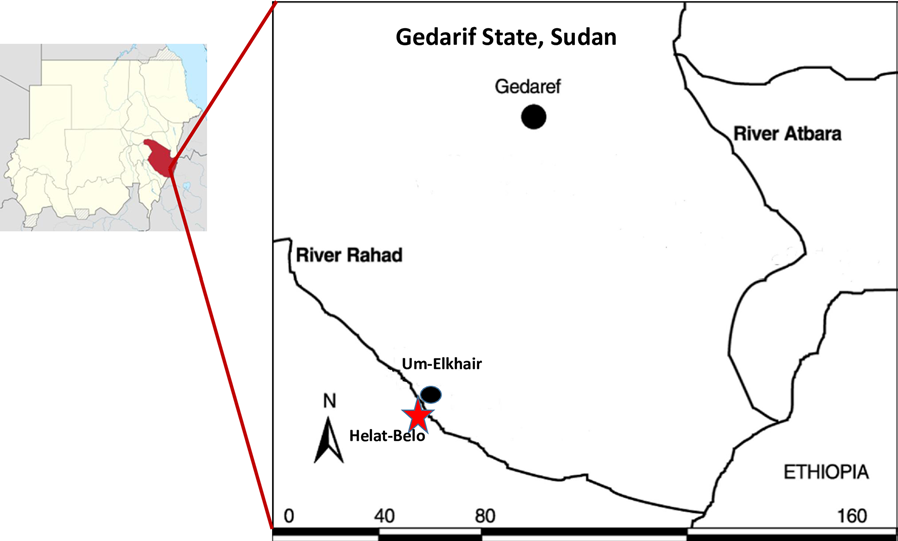
Map showing location of study area around Belo village, Gedarif state, Sudan

The study was conducted during three consecutive sand fly seasons, March-June 2016–2019, in the area of Belo Village (Nour-Elmadina) that is located on the south-western bank of River Rahad (12°52′476″N, 035°09′039″E), approximately 100 km south west of Gedarif town, Gedarif state, eastern Sudan (Fig. 1).

The ecology of the area was described in previous publications [6, 18]. The soil is mainly black cotton soil (chromic vertisol), with mixed stretches of sand fly/clay soil (locally called “Azaza”) in some locations inside the village and near the river. *Balanites aegyptiaca* and *Acacia seyal* trees dominate the natural vegetation. Dense forests are found around the village at the east, south, and western sides about 5 km from Dinder National Park. Citrus and neem (*Azadirachta indica*) are found near the riverbank and inside the village, respectively.

The climate is tropical continental with an annual rainfall of 800 mm. The year is divided into three main seasons; a rainy relatively cooler season (autumn: June-October), a dry relatively cool season (winter: November-February) and a dry hot season (summer: March–May). The mean maximum daily temperature varies from 30.0 °C in August to 42.0 °C in April; while the mean minimum daily temperature ranges between 20.0 °C in January and 32.0 °C in April-May. Wind blows from the north during the dry season (November–May) and from the south during the rainy season (June–October). All experiments described in this study were conducted on calm, windless nights, in a small plot of land (approximately 200 × 200 m) located at the southern edge of Belo village.

The inhabitants of the village belong to the Fulani tribe. Most of the population lives in huts constructed of thatched grass or mud that are surrounded by a straw fence. The villagers raise large numbers of goats, sheep, cattle and chickens. For religious reasons, the inhabitants do not keep dogs anywhere in the village and cats are rarely seen.

### Comparison of numbers of *P. orientalis* captured on differently coloured sticky traps under variable lunar light illumination

We conducted four field experiments in March–April 2016, March–June 2017, and April–June 2018 to compare numbers of *P. orientalis* captured on differently coloured sticky paper traps. Each trap was prepared by coating a coloured A4 (12.0 × 29. 7 cm) paper or A4 transparent plastic sheet (12.0 × 29. 7 cm) with pure sesame oil, purchased at a bookstore in Khartoum, Sudan. No information was available about the company of production or the specifications of the A4 papers

### Experiment 1: preliminary investigation on efficacy of vertical sticky coloured traps in capturing *P. orientalis* under quarter-lunar and half-lunar light conditions

**Fig. 2:**
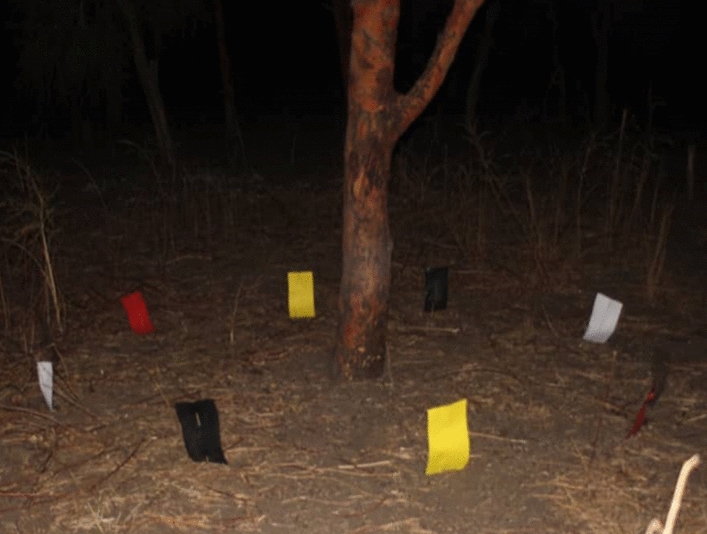
Set-up of an experiment comparing number of sand flies captured on differently coloured sticky traps. Traps were set vertically around an *Acacia seyal* tree on a black cotton soil site at the periphery of Belo village, Gedarif state, eastern Sudan; March–April 2016. The experiments were conducted on calm nights, without noticeable wind blowing from any direction. The circular design of traps setup was intended to ensure that they had similar exposure to sand flies arriving from the surroundings. Each circle contained a duplicate of black, red, yellow, white and transparent traps (located between the red and the white traps)

In March-April 2016, we conducted a preliminary proof of concept experiment to compare numbers of *P. orientalis* captured on 5 differently coloured vertical sticky traps set under quarter-lunar-half lunar light conditions. A duplicate of 5 sets of sticky traps made of transparent plastic sheets and black, red, yellow, and white papers were placed vertically on wooden sticks at 3 cm above the ground in a 2–5 m diameter circle on the ground between 18:00–06:00 h (Fig. 2). The circular setup of the experiment was chosen to allow all traps equal exposure to sand flies arriving from the surrounding. Due to our uncertainty of the best trapping spots that produce adequate numbers of *P. orientalis*, we replicated the experiment simultaneously at 4 different microhabitats identified in the study site. Thus, the coloured sticky traps were set out around an *A. seyal* tree, a *B. aegyptiaca* tree, a termite mound, and an open space between the trees. At each microhabitat type we made 5 replicates of the circular experimental unit; thus resulting in 10 traps for each of the colour for each of the 4 different microhabitats. The experiment was repeated for 6 nights in April 2016 that spanned the period when the moon was quarter lunar to half lunar. Sand flies collected in each sticky trap were picked up by a fine brush and processed as described below. Samples from different circles were pooled according to the colour of the trap and the collection site. The total collection of *P. orientalis* from each 10 sticky traps of a specific colour and specific site represented a trap night.

### Experiments 2 and 3: comparison of numbers of *P. orientalis* captured on differently coloured sticky traps set vertically, under full lunar and dark night conditions

In the second and the third experiments we compared the numbers of *P. orientalis* captured on different vertical coloured traps set under either full lunar (experiment 2: 13–16th of the lunar month in April 2017) or full dark nights (experiment 3: 27–29th and the first night of two lunar months in March and June 2017). The trapping setup of these experiments followed the same circular design described above. However, we added blue and green sticky traps to the comparison. To keep the same circle diameter, the number of traps from each colour was reduced to one specific colour trap per circle. Trapping was done for 4 dark nights and 8 lunar nights. Each night, a total of 10 circles each containing one trap of each colour, were set between 18:00–06:00 h in one site with mixed *A. seyal* and *B. aegyptiaca* trees at the periphery of Belo village. Sand flies were collected and processed as described above. Sand flies from all 10 sticky traps for each colour per night were pooled as a single trap night sample.

### Experiment 4: comparison of numbers of *P. orientalis* captured on differently coloured sticky traps set horizontally, under dark night conditions

The 4th experiment was carried out in April 2018, to compare numbers of *P. orientalis* captured on differently coloured traps when placed horizontally on the ground surface. The experiment was conducted on full dark nights in April 2018, using the circular experimental design described above; except for the spatial orientation of the traps, which were placed horizontally on the ground, supported in place by small stones. Black A4 papers were not available during the time of this experiment and therefore this trap colour was not included in the comparisons, which were made between the red, transparent, white, yellow, green and blue traps. A total of 5 replicates were conducted in 3 nights. Sand flies were processed and recorded as described above.

### Comparison of numbers of *P. orientalis* captured on vertical and horizontal white sticky traps

We examined whether placing sticky traps in a vertical position to intercept a possible sand fly path would result in capturing higher numbers of *P. orientalis* than traps placed horizontally on the ground. In April 2017, 10 sets of traps, each consisting of one horizontal and one vertical white sticky traps, spaced at ½ m apart, were placed in the study site described above, overnight from 18:00–06:00 h. By next morning, sand flies on the traps were picked up and processed for subsequent identification as described above. Results were recorded as numbers of *P. orientalis* per trap night, which is defined as total number of individuals per 10 horizontal or 10 vertical traps, per night. The experiment was replicated 6 times during dark night conditions. The numbers of *P. orientalis* per horizontal or vertical trap nights were compared using the statistical tests described below.

### Effects of sticky traps heights on their efficacy in capturing *P. orientalis*

An experiment was done during full dark nights in April 2018 and March 2019 to compare the collection of *P. orientalis* on 3 sets of white sticky traps placed horizontally on the ground surface, horizontally on 15 cm height metal stools, or vertically on small sticks as described above. Each set consisted of 10 traps, placed ½ m apart in a straight line. To allow similar access to the surrounding area, the three lines of traps were set out in a triangle shape. Trapping was continued from 18:00 to 06:00 h. The experiment was replicated 4 times in March 2018 and 9 times March 2019. The positions of the three sets of traps were rotated with each new replicate, so that each trap received equal exposure to the surroundings. Sand flies found in each set of traps were collected, sorted out and identified, and then compared as described above.

### Observations on mating behaviour of sand flies as they land on non-sticky white papers placed horizontally on the ground

We conducted preliminary field observations to determine whether the bright surface of a non-sticky untreated paper provides an arena for courtship and mating of *P. orientalis.* Two new non-sticky clean white A4 paper were placed on the ground at sunset and used to observe whether both male and female *P. orientalis* (and other sand flies) land and copulate on its surface. To avoid disturbing the sand flies during our observations, we had to stay about 1 m away from the paper and came close only when sand flies landed and/or started copulation. No specific protocol was followed to document the behaviour of the sand flies. The observations were repeated for 4 consecutive evenings for 30 min at sunset. We made several attempts to capture the adult sand flies for confirmation of identification and counting, but none were successful.

### Sand fly preservation and identification

Sand flies captured on the sticky paper traps were removed using small fine brushes; washed in a detergent and water, and finally preserved in 70% ethanol for subsequent processing. Preserved sand flies were sorted by sex and genus under a binocular dissecting microscope. *Sergentomyia* sand flies were counted without further processing. *Phlebotomus* sand flies were mounted in PVA medium (BioQuip, Rancho Dominguez, CA, USA) and then identified to the species level, using standard morphological features described in relevant taxonomic keys [8, 13, 19, 20].

### Statistical analysis

Data were analyzed using SPSS statistical package (version 24; SPSS Inc., Chicago, IL, USA). The data were not normally distributed. Therefore, the non-parametric Kruskal-Wallis test, Mann-Whitney U-test and Wilcoxon signed-rank test were used to compare the median numbers of sand fly specimens attracted to differently coloured sticky paper traps, at variable lunar conditions and spatial orientations. For experiments 1–4, we adjusted all probability levels of significant differences in pairwise tests with the Bonferroni correction for multiple comparisons. The Wilcoxon’s signed-rank test for paired data was used to analyze the results of the last two experiments, which were done to compare numbers *P. orientalis* captured on sticky traps set at different spatial orientations and heights. The data from these two experiments were considered paired at each trapping event due to the logical connection between the performance of different traps placed at the same night, location and environmental conditions. Sample size limitations did not allow us to conduct multivariate analysis to examine interaction of different conditions on efficacy of sticky paper traps.

## Results

### Species and sex composition of sand flies collected during the study

During the whole study, a total of 12495 sand flies were captured on sticky traps. The collection consisted of *P. orientalis* (2403, 19.2%), *Sergentomyia spp.* (9840, 78.8%), and small numbers of *P. papatasi* sand flies (252, 2.0%). We did not conduct species identifications for the *Sergentomyia* sand flies, which we grouped together as total numbers of males and females. Most of the collection of *P. orientalis* consisted of males (total = 2196, 91.4%), and significantly smaller number of females (total = 207, 8.6%) (*U* = 8477.5, *df* = 1, *Ζ* = -10.170, *P* < 0.001).

On the commencement of the study, in March-April 2016, we attempted to maximize the chances of successful collection of *P. orientalis* by replicating the vertical coloured traps experiment in the 4 microhabitats that we could identify in the study site. Analysis of the results of this experiment, however, showed no significant differences between numbers of *P. orientalis* captured on traps set around *A. seyal*, *B. aegyptiaca*, termite mound, or on the open space between the trees (Kruskal-Wallis test: *χ*^2^ = 3.582, *df* = 3, *P* = 0.311, *n* =113 for all coloured traps; *χ*^2^ = 3.671, *df* = 3, *P* = 0.299, *n* = 23 for black traps; *χ*^2^ = 0.238, *df* = 3, *P* = 0.971, *n* = 23 for red traps; *χ*^2^ = 1.955, *df* = 3, *P* = 0.582, *n* = 23 for transparent traps; *χ*^2^ = 0.792, *df* = 3, *P* = 0.792, *n* = 22 for white traps; *χ*^2^ = 0.988, *df* = 3, *P* = 0.804, *n* = 22 for yellow traps). Therefore, in subsequent work, we conducted all experiments in one site, as described above.

### Efficacy of differently coloured vertical sticky traps in collection of *P. orientalis* under variable lunar light conditions

**Fig. 3:**
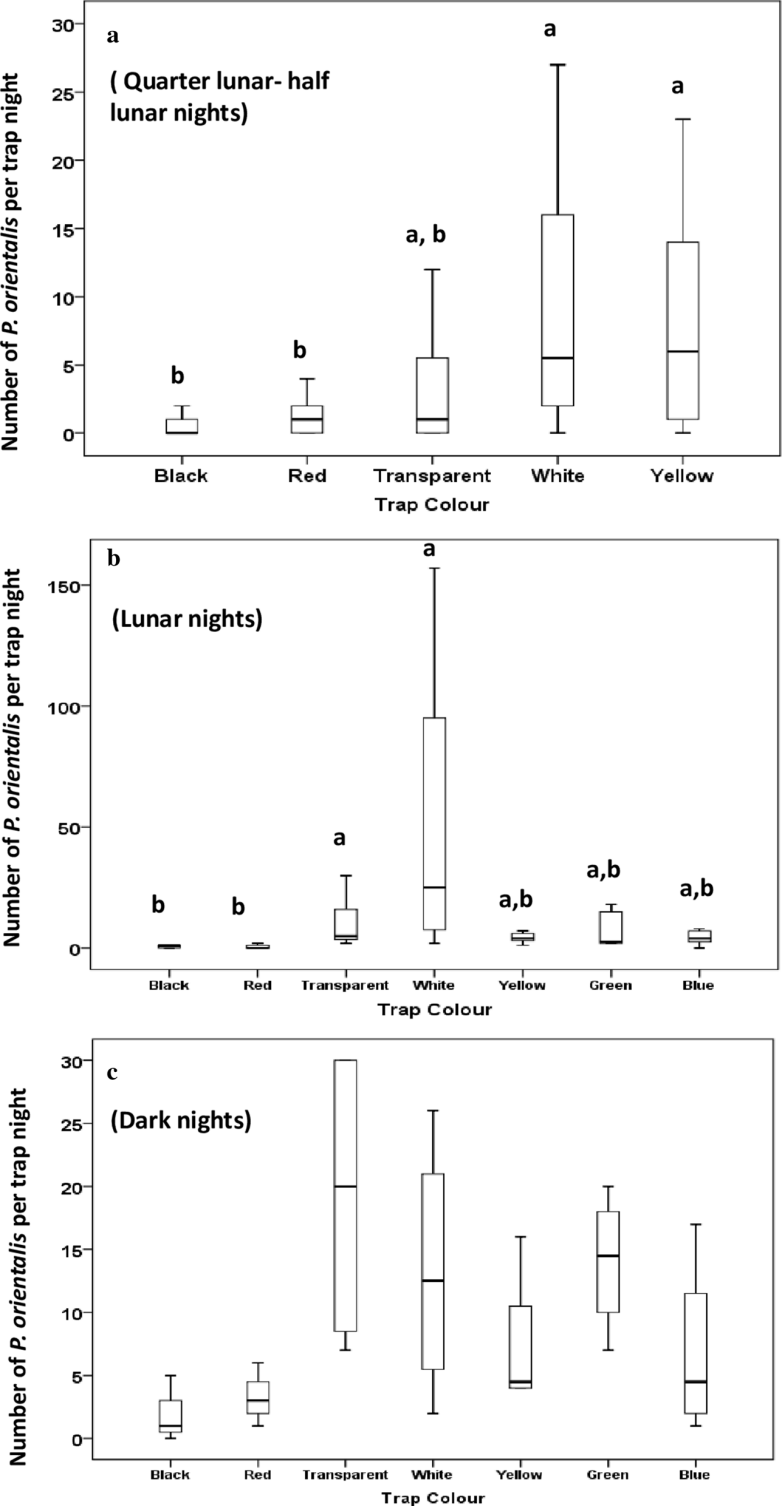
Numbers of *Phlebotomus orientalis* sand flies captured on coloured sticky traps, during different lunar phases. Each trap night is the total number of male and female sand flies captured on 10 A4 size papers or transparent plastic sheets, placed vertically on the ground, between 18:00–06:00 h at different microhabitats in Belo village, Rahad region, eastern Sudan. Boxplot boxes represent the interquartile range, the horizontal lines represent the medians and the upper and lower whiskers represent the maximum and minimum values. **a** Quarter lunar-half lunar nights (April 2016). **b** Full-lunar nights (March–June 2017). **c** Full dark nights (April 2017). Note the different scales used for Y-axes. Different letters above bars indicate significantly different values (Kruskal-Wallis test, followed by pairwise analysis, adjusted by Bonferroni correction for multiple comparisons)

Under all lunar light conditions, there were significant variations in numbers of *P. orientalis* captured on differently coloured vertical sticky-paper traps (Fig. 3a-c; *χ*^2^ = 29.48, *df* = 4, *P* < 0.001 for quarter-half lunar conditions in experiment 1; *χ*^2^ = 30. 957, *df* = 6, *P* < 0.001 for lunar conditions in experiment 2; *χ*^2^ = 14.705, *df* = 6, *P* = 0.023 for dark nights in experiment 3). The relative performance of the differently coloured traps varied from one experiment to another, depending on the lunar light conditions. However, in all experiments, the black and the red traps captured similarly lower numbers of *P. orientalis* compared to other coloured or white traps (Additional file 1: Table S1–S4).

In the preliminary experiment, carried out under quarter to half lunar light conditions, the white and yellow sticky traps showed the highest efficacy in trapping *P. orientalis* (white: median = 5.5, IQR: 1.5–6.0 and yellow: median = 6.0, IQR: 1.0–14.3). The similar numbers of *P. orientalis* captured on each of these traps (*Z* = 0.909, *df* = 1, *P* = 1.000) were significantly higher than those captured on the black: median = 0.0, IQR: 0.0–1.0) and red traps: median = 1.0, IQR: 0.0–2.0; *Z* = 39.725, *df* = 1, *P* < 0.001 for white *vs* black; *Z* = 38.816, *df* = 1, *P* < 0.001 for yellow *vs* black; *Z* = 29.419, *df* = 1, *P* = 0.014 for white *vs* red and *Z* = 28.240, *df* = 1, *P* = 0.020 for yellow *vs* red. On the other hand, the transparent traps captured intermediate numbers of *P. orientalis* (median = 1, IQR: 0.0–5.8), which were statically similar to the numbers found on other coloured traps (Additional file 1: Table S1).

On full lunar nights, the white and transparent sticky traps captured the highest numbers of *P. orientalis* (white: median = 25.0, IQR: 5.8–11.0; transparent: median = 5.0, IQR: 3.3–19.0, Fig. 3b). These traps significantly outperformed the black and red traps (black: median = 1.0, IQR: 0.0, 1.0 and red: median = 0.0, IQR: 0.0–1.0, Additional file 1: Table S2). On the other hand, yellow, green and blue traps set at the same conditions appeared to capture intermediate numbers of *P. orientalis* (yellow: median = 4.0, IQR: 2.0–6.5; green: median = 2.5, IQR: 2.0–15.8; blue: median = 4.0, IQR: 1.8–7.5), which were similar to both the high numbers on the white-transparent traps and the low numbers on the red-black traps (Additional file 1: Table S2, Fig. 3b).

On full dark nights, the transparent, white, green, yellow and blue traps appeared to capture higher numbers of *P orientalis* than black and red traps (transparent: median = 20.0, IQR: 7.8–30.0; white: median =12.5, IQR: 3.8–23.5; green: median = 14.50, IQR: 8.5–19.0; yellow: median = 4.5, IQR: 4.0–13.3 and blue: median = 4.5, IQR:1.5–14.3; black: median = 1.0, IQR: 0.3–4.0 and red: median = 3.0 IQR: 1.5–5.3). However, none of the pairwise differences were significant, when adjusted for multiple comparisons (Additional file 1: Table S3; Fig. 3c).

### Numbers of *P. orientalis* captured on horizontal sticky coloured traps in full dark nights

**Fig. 4:**
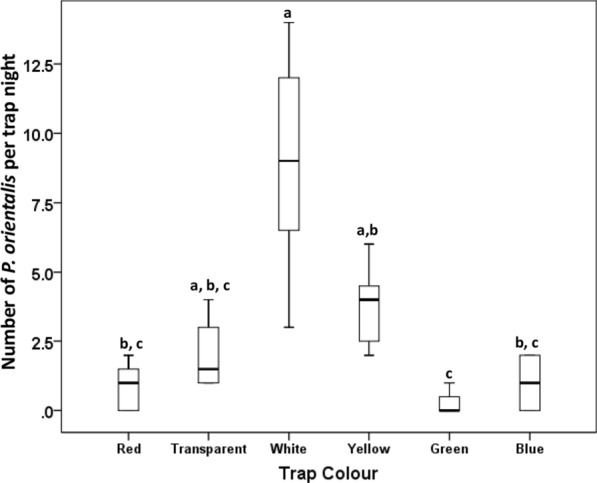
Numbers of *Phlebotomus orientalis* sand flies captured on horizontal coloured sticky traps, during full dark nights. Each trap night reflects the number of sand flies found on 10 A4 size paper or a transparent plastic sheets placed horizontally on the ground, between 18:00–06:00 h at different microhabitats at Belo village, Rahad region, eastern Sudan (March–June 2018). Boxplot boxes represent the interquartile range, the horizontal lines represent the medians and the upper and lower whiskers represent the maximum and minimum values. Different letters above bars indicate significantly different values (Kruskal-Wallis test, followed by pairwise analysis, adjusted by Bonferroni correction for multiple comparisons)

When we repeated the sand fly collection using horizontal traps under dark night conditions, we encountered strong significant variations in numbers of *P. orientalis* captured on differently coloured sticky papers (*χ*^2^ = 35.017, *df* = 5, *P* < 0.001; Fig. 4, Additional file 1: Table S3). As observed above for quarter-half lunar and full lunar nights, significantly higher numbers of *P. orientalis* were captured on the white and yellow traps (white: median = 9.0, IQR: 5.8–12.5 and yellow: median = 4.0, IQR: 2.3–4.80) than the transparent, green, blue and red traps (transparent: median = 1.5, IQR: 1.0–3.0; green: median = 0.0, IQR: 0.0 –0.8; blue: median = 1.0, IQR: 0.0–2.0 and red: median = 1.0, IQR: 0.0–1.8).

### Comparison of sex ratio of *P. orientalis* captured by differently coloured traps under variable lunar light conditions

Under all night lunar conditions and trap spatial orientations, sticky traps of all colours captured higher numbers of male *P. orientalis* than females (Table 1). Due to zero records of females, we were not able to calculate the sex ratios for black and red traps under quarter lunar - half lunar and full lunar conditions, respectively. We also excluded the 1:1 sex ratio of the green trap under dark conditions, due to the fact that only one male and one female were collected in all 8 replicates.

**Table 1:** Comparison of total numbers and sex ratio ratios of *Phlebotomus orientalis* sand flies captured by coloured sticky traps (STs) under different lunar light conditions and traps orientations in Belo Village (Gedarif state, Sudan; April–June 2016–2018)

Trap colour	*n*^a^	Total no. of sand flies	No. of males	No. of females	Male:Female^b^ sex ratio
Experiment 1: Performance of vertical coloured STs under quarter-half-lunar light conditions					
Black	18	6	6	0	na
Red	21	23	22	1	22:1
Transparent	20	62	58	4	15:1
White	22	182	174	8	22:1
Yellow	22	166	155	11	15:1
Total	103	439	415	24	17:1
Experiment 2: Performance of vertical STs traps under full lunar light conditions					
Black	8	5	3	2	2:1
Blue	8	35	32	3	11:1
Green	6	42	40	2	20:1
Red	7	4	4	0	na
Transparent	8	81	74	7	11:1
White	8	414	408	6	68:1
Yellow	5	21	18	3	6:1
Total	50	602	579	23	25:1
Experiment 3: Performance of vertical coloured STs under dark night conditions					
Black	4	7	6	1	6:1
Blue	4	27	25	2	13:1
Green	4	56	46	10	5:1
Red	4	13	11	2	6:1
Transparent	4	77	63	14	5:1
White	4	53	46	7	7:1
Yellow	4	29	27	2	14:1
Total	28	262	224	38	6:1
Experiment 4: Performance of horizontal coloured STs under dark night conditions					
Blue	8	8	6	2	3:1
Green	8	2	1	1	na
Red	8	7	5	2	3:1
Transparent	8	16	11	5	2:1
White	8	72	47	25	2:1
Yellow	8	30	21	9	2:1
Total	48	135	91	44	2:1

^a^n, number of replicates. Note that the number of replicates has a slight variation, due to missing data

^b^na, no sex ratios were calculated due to presence of a zero record or too small number of males and females

No statistical analysis was conducted to compare the sex ratios of *P. orientalis* captured on differently coloured traps. However, it was clear that male biased sex ratios were highest on vertical traps under lunar nights (25:1 for all traps), followed by vertical traps under quarter-lunar nights (17:1), vertical traps under dark night conditions (6:1), and lastly horizontal traps under dark night conditions (2:1). Moreover, the sex ratios of *P. orientalis* captured on different vertical coloured traps appeared to vary with lunar light conditions. On quarter-lunar-half lunar nights, the highest male biased ratios were seen on red and white traps (22:1 and 21:1, respectively). On full lunar nights, these ratios appeared to be exceptionally high on white traps (68:1) followed by green traps (20:1). On dark nights, the highest male:female sex ratios were recorded from yellow and blue traps (14:1 and 13:1, respectively). In contrast to the vertical traps, trap colour did not seem to affect the male-biased sex ratios of *P. orientalis* captured on horizontal traps, which ranged between 2:1 for the white, yellow and transparent traps to 3:1 on the blue and red trap (Table 1).

### Effects of spatial orientations of white sticky traps on their efficacy in capturing *P. orientalis* sand flies

**Fig. 5:**
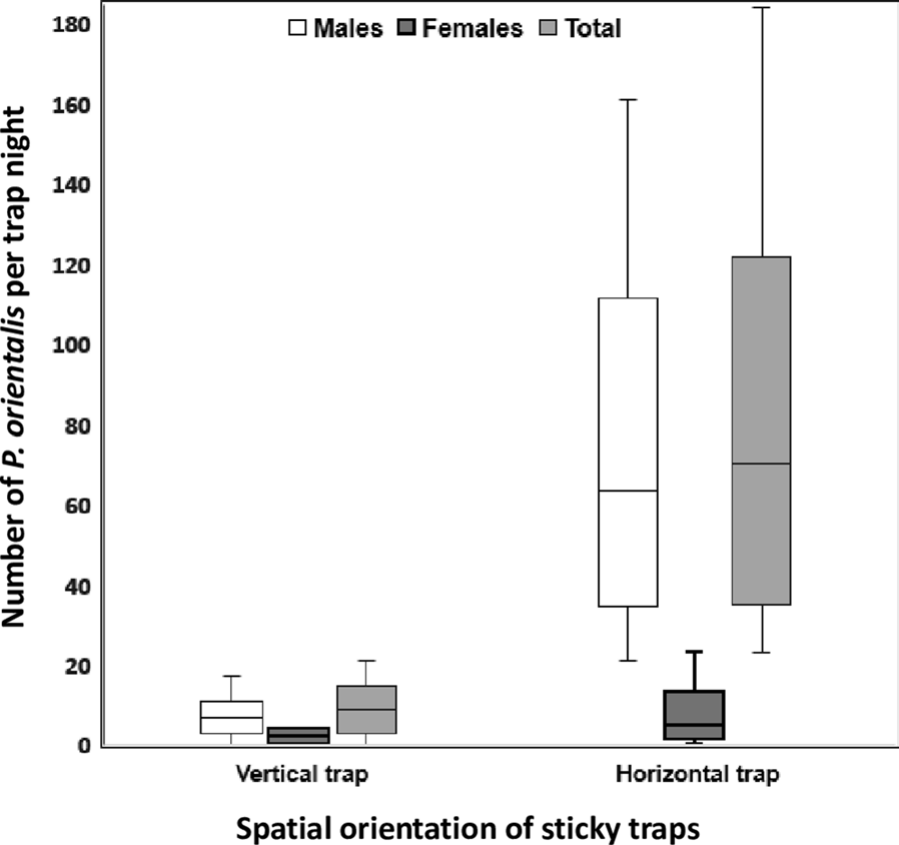
Comparison of numbers of *Phlebotomus orientalis* sand flies captured on vertical and horizontal white sticky traps. Each trap night reflects the number of sand flies captured on 10 A4 size paper traps placed between 18:00–06:00 h at the periphery of Belo village, Gedarif state, eastern Sudan (April 2017). Boxplot boxes represent the interquartile range, the horizontal lines represent the medians and the upper and lower whiskers represent the maximum and minimum values

The second set of experiments, testing the hypothesis that sticky paper traps are more attractant-based than interception-based sampling tools, compared the trapping efficacy of adjacently placed A4 white sticky paper traps positioned in horizontal spatial orientation on the ground *versus* traps placed vertically on the ground as described above. Significantly higher numbers of *P. orientalis* were captured on the horizontal traps than on the vertical traps (Fig. 5; *Ζ* = 2.201, *df* = 1, *P* = 0.028), resulting in an 8-fold increase when the traps were placed horizontally on the ground surface (median = 70.0, IQR: 35–122) *versus* when traps were placed vertically on the ground (median = 9.0, IQR: 3–15). On both horizontal and vertical traps there were more males than female *P. orientalis* (sex ratio = 10:1 for horizontal traps and 4:1 for vertical traps).

### Effects of sticky trap heights from the ground on their efficacy in capturing *P. orientalis* sand flies

**Fig. 6:**
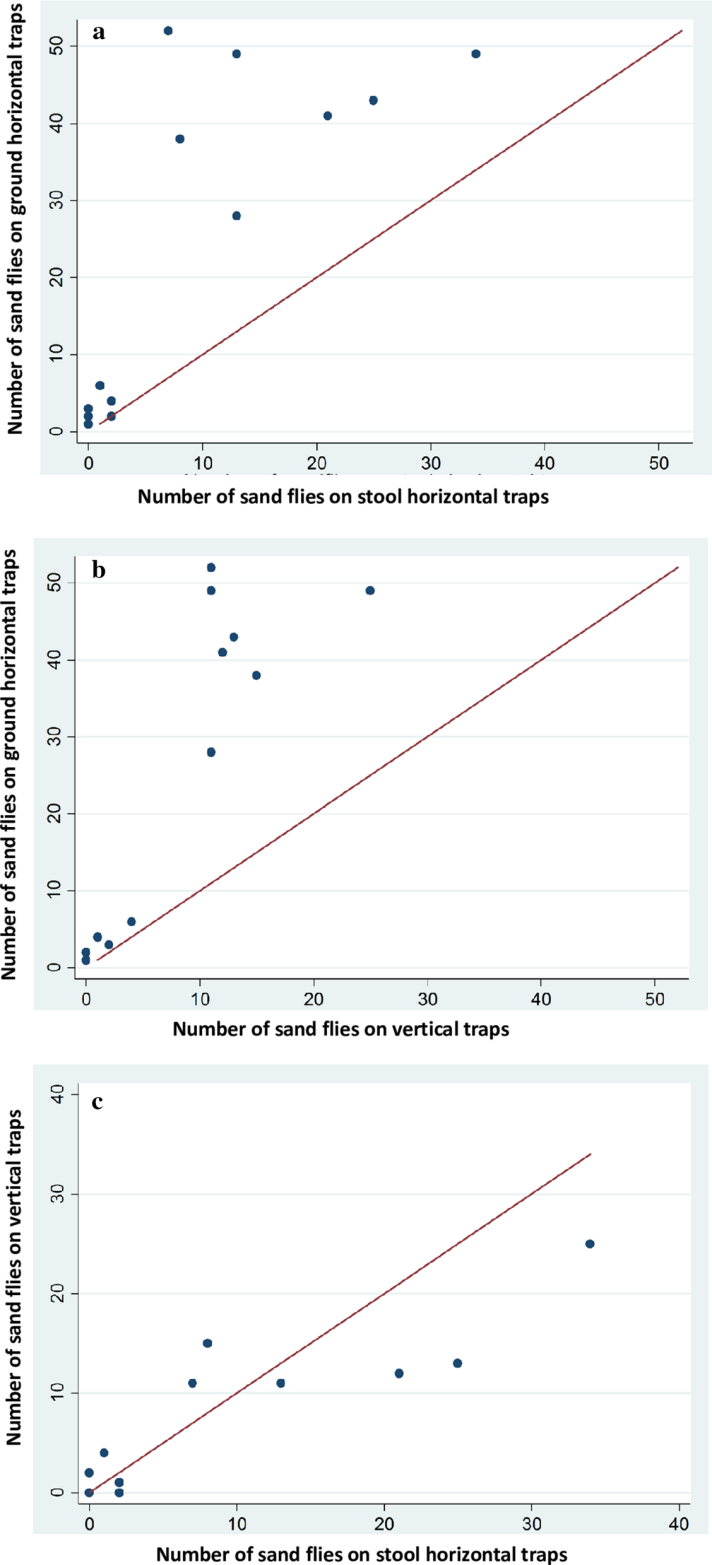
Numbers of *Phlebotomus orientalis* captured on sticky traps placed at different heights and spatial orientations. The three scattered diagrams compare numbers of *P. orientalis* captured on the following sticky trap setups. **a** Horizontal traps placed on 15 cm height stools *vs* horizontal traps placed on the ground. **b** Vertical traps placed on the ground *vs* horizontal traps placed on the ground. **c** Horizontal traps placed on the stools *vs* vertical traps placed on the ground. Note that in **a** and **b**, all points fell above the 45° diagonal line, showing that in every replicate the horizontal ground traps captured higher *P. orientalis* than the horizontal stool traps and the vertical traps. In contrast in **c**, the 45° reference line straddled most data points, indicating similar numbers of *P. orientalis* on the vertical traps and the horizontal stool traps

**Fig. 7:**
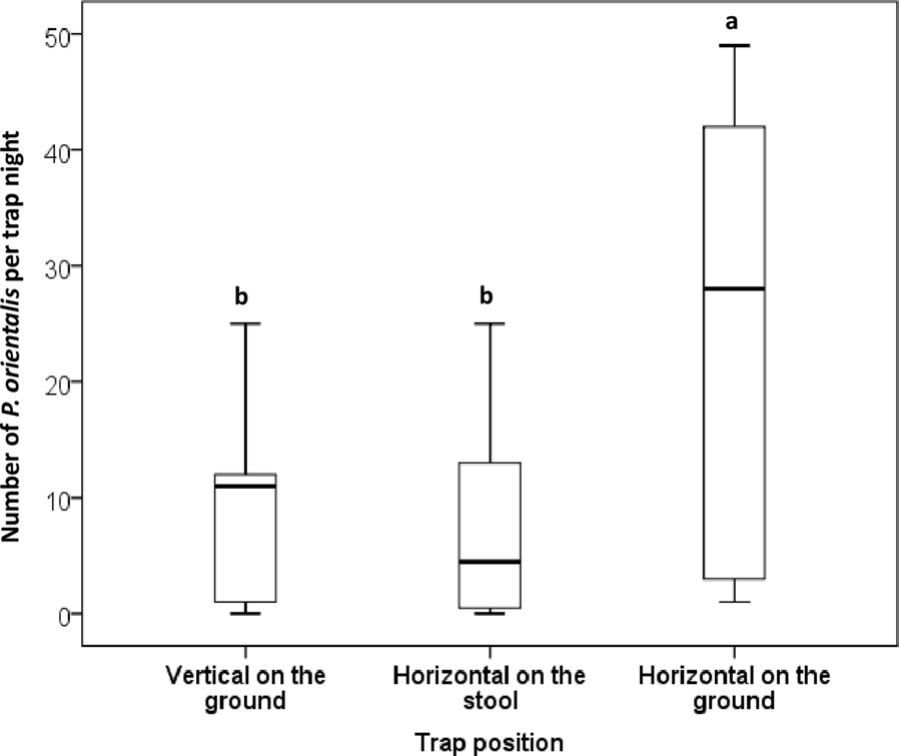
Effects of spatial orientation and height on collections of *Phlebotomus orientalis* sand flies by sticky traps. Experiment conducted in April 2018 in Belo village, eastern Sudan. Boxplot boxes represent the interquartile range, the horizontal lines represent the medians and the upper and lower whiskers represent the maximum and minimum values. Different letters above bars indicate significantly different values (Wilcoxon’s signed-rank test)

In a subsequent experiment, we examined whether differences seen in the performance of horizontal and vertical traps were due to height from the ground level or actual spatial orientation of the trap. Therefore, we compared numbers of *P. orientalis* captured on three sets of traps, placed horizontally on the ground, horizontally on 15 cm metal stools or vertically on the ground. The horizontal trap placed on the ground captured significantly higher numbers of *P. orientalis* (median = 28, IQR: 3–43) than the horizontal traps placed on the stool (median = = 7, IQR: 1–13; *Z* = 3.062, *df* = 1, *P* = 0.002) and the vertical traps (median = 11, IQR: 1–12; *Z* = 3.185, *df* = 1, *P* = 0.001) (Figs. 6, 7). To the contrary, there were no significant differences between numbers of *P. orientalis* captured on the horizontal stool trap and the vertical traps (*Z* = 0.760, *df* = 1, *P* = 0.447). Interestingly, during sample processing we noticed that most of the flies captured on the vertical traps were found on the edge of the paper close to ground level. We did not quantify this observation, which was evident on most vertically placed traps.

### Observations on courtship and mating behaviour of *P. orientalis* when landing on untreated white papers placed on the ground

We noticed a characteristic behaviour of male and female sand flies that landed on the white non-sticky papers. The males arrived and landed readily on all white papers as we placed them on the ground. Within a few minutes, female sand flies started to follow the males in landing on the paper, and began to clearly copulate with them. This behavioural pattern occurred on all papers that we placed on the ground and continued throughout the 30 min observation period. At each occasion, the sand fly males landed before the females and performed a distinct behavior; running in straight lines on the paper. Due to a lack of suitable instruments, we were not able to quantify the details of these behavioural events. We were also unable to capture the sand flies for species identification. However, relying on prior experience of sand fly fauna in the area and the predominance of *P. orientalis* in the *Phlebotomus spp* collection, we could judge that many of the female and male sand flies that we observed mating on the papers were probably *P. orientalis*.

## Discussion

Properly designed traps are important tools for the sampling of disease vectors, studying their biology and ecology, and evaluation of vector control operations. Understanding the mechanism by which these traps capture the target vector species is key to successful optimization of their performance and interpretation of their results. To date, sand fly traps have been classified under two broad categories: interceptive tools (e.g. sticky traps) and attractant-based tools (e.g. CDC light traps) [12]. While the interception traps capture active sand flies in a given habitat without bias, attractant-based tools lure sand flies by specific physical or chemical cues, such as light, CO_2_, or host odors and kairomones [16, 21–25].

Sticky traps have been widely viewed as interceptive sampling tools and therefore in most studies they were intentionally placed in vertical positions to intercept a presumed sand fly flight path [12]. The results of our current study contradict this notion strongly; and show that for *P. orientalis*, the sticky traps are by far more attractant-based than interception-based sampling tools. First, in the coloured sticky traps experiment, if the traps capture *P. orientalis* by interception, collection on differently coloured papers should be distributed evenly. It must be emphasized that in this experiment we placed the coloured sticky papers on the vertical position to maximize any interception that might have existed. Clearly, interception was not the mechanism of trapping since the vector showed strong differential attraction to specific colours, with negligible numbers of flies captured on the black and red traps. Given that black-colored objects do not reflect light and red light elicits minimum visual sensitivity by insects, this diminished collection of *P. orientalis* on the black and red sticky traps provides a strong argument for active attraction. Secondly, an interception mechanism implies that vertical placement of the sticky traps in the flight path of *P. orientalis* should result in better trapping efficacy than the horizontal orientation. Of note, the results of the experiment that compared the efficacy of horizontal and vertical traps actually showed the completely opposite findings. We observed an 8-fold increase in the collection of *P. orientalis* on horizontal compared to vertical traps.

Overall, the comparison of numbers of *P. orientalis* captured on differently coloured sticky traps showed that the vector was more attracted to the white, yellow, transparent and green traps than to the blue and red traps. As shown previously for CDC traps with standard incandescent light [26], it seems that lunar cycle can influence the performance of differently coloured traps. More numbers of *P. orientalis* were attracted to the white and yellow vertical traps during quarter lunar, half lunar and full lunar nights while in full dark there was no significant difference in the performance of differently coloured traps. These results indicate that the differential attraction of bright colour vertical traps is higher under more lunar illumination. However, the absence of significant difference in the performance of differently coloured traps under dark night conditions may be due to the small number of replicates done in this experiment. Interestingly, the spatial orientation of the traps seemed to alter the differential attraction of *P. orientalis*, since in dark nights white and yellow horizontal traps attracted significantly more numbers of the vector than all other coloured traps. Thus, it seems that variations of the intensity of reflected light that results from the lunar cycle and the trap position can influence the observed colour-based attraction of *P. orientalis*.

To the best of our knowledge, this is the first published report of differential attraction of sand flies to coloured sticky paper traps. Nonetheless, the results correspond to the findings of Burkot et al. [21], who reported that while sticky traps baited with chemical light sticks in the visible spectrum captured significantly more sand flies than infrared baited sticky traps and un-baited sticky traps, there was no significant difference in the efficacy of traps baited with differently coloured light sticks. It must be stressed that no species identification was done on the sand flies caught by these authors; a problem that seriously compromises the results and the conclusion of this study. On the other hand, a number of field and laboratory studies demonstrated that sand flies have true colour vision and could be differentially attracted to specific wavelengths in the UV and blue-green-yellow region [27, 28]. Similarly, studies show that sand flies are more attracted to CDC traps baited with UV light than standard CDC traps baited with incandescent light [21] and their attraction may also be influenced by the intensity of the light [27–29]. It must be stressed that in our study we had no means to control for or measure light intensity other than conducting the experiments under low intensity (dark lunar nights) and higher intensities (full lunar nights).

Despite the variations in collection during different lunar phases, there was an overall higher attraction of *P. orientalis* to white papers than other coloured traps. This preference was probably due to the fact that the light reflected by white-coloured paper is brighter and contains the full spectrum of light. It is also probable that the white Xerox paper used in the study may reflect more UV light which is known to attract sand flies, other Dipterans and many other insects [30]. It is common practice by manufacturers to add UV-reflecting chemicals to white Xerox paper to brighten its appearance. Cronin & Bok [31] suggested that most insect species include UV wavelength in their range of spectral sensitivity because of its adaptive ecological value as a signal of potential sources of sugar, intraspecific communication, and orientation during flight. Therefore, we recommend that further optimization of the sticky traps for routine surveillance of *P. orientalis* should be based on the white traps without any change in colour.

The results of the experiment comparing efficacy of vertical and horizontal traps correspond closely to the findings of Gebresillassie et al. [7], who reported that, in agricultural fields of NW Ethiopia, horizontally placed A4 size sticky paper traps captured significantly higher numbers of *P. orientalis* than similar traps that were hung vertically at 30 cm above the ground. However, the differences between horizontal and vertical traps detected in this previous study could also be due to elevation from the ground where more sand fly flight activity takes place [11]. The results of current experiments on efficacy of vertical and horizontal traps also support the findings of Moncaz et al. [16]. These authors showed that un-baited large horizontal traps (LHT) with sticky surfaces made of 60 × 60 cm white polypropylene boards coated with sesame oil and placed on a metal frame 15 cm above the ground captured large numbers of male *P. orientalis* [16]. Although the experiment conducted by these authors did not include direct comparison with vertical traps, the large numbers of *P. orientalis* captured on these LHT stand out as evidence of attraction, especially when compared to literature reports of traps laid on the vertical axis.

To achieve further progress towards optimization of the sticky traps as standard tools for sampling *P. orientalis*, we compared numbers of *P. orientalis* captured on traps set vertically on the ground, or horizontally on the ground or horizontally on stools set 15 cm above the ground. The data confirmed the above findings that horizontal sticky traps attract higher numbers of *P. orientalis* than vertical traps. Interestingly, the results showed that when traps were raised horizontally on slightly elevated stools, they had no advantage over vertical traps; thus indicating location on the ground as a main component of the attraction of the horizontal traps to the vector. Based on these results, it is recommended that in routine surveillance of *P. orientalis* the standard sticky traps should be placed horizontally on the ground instead of being laid on stools or positioned vertically.

**Fig. 8:**
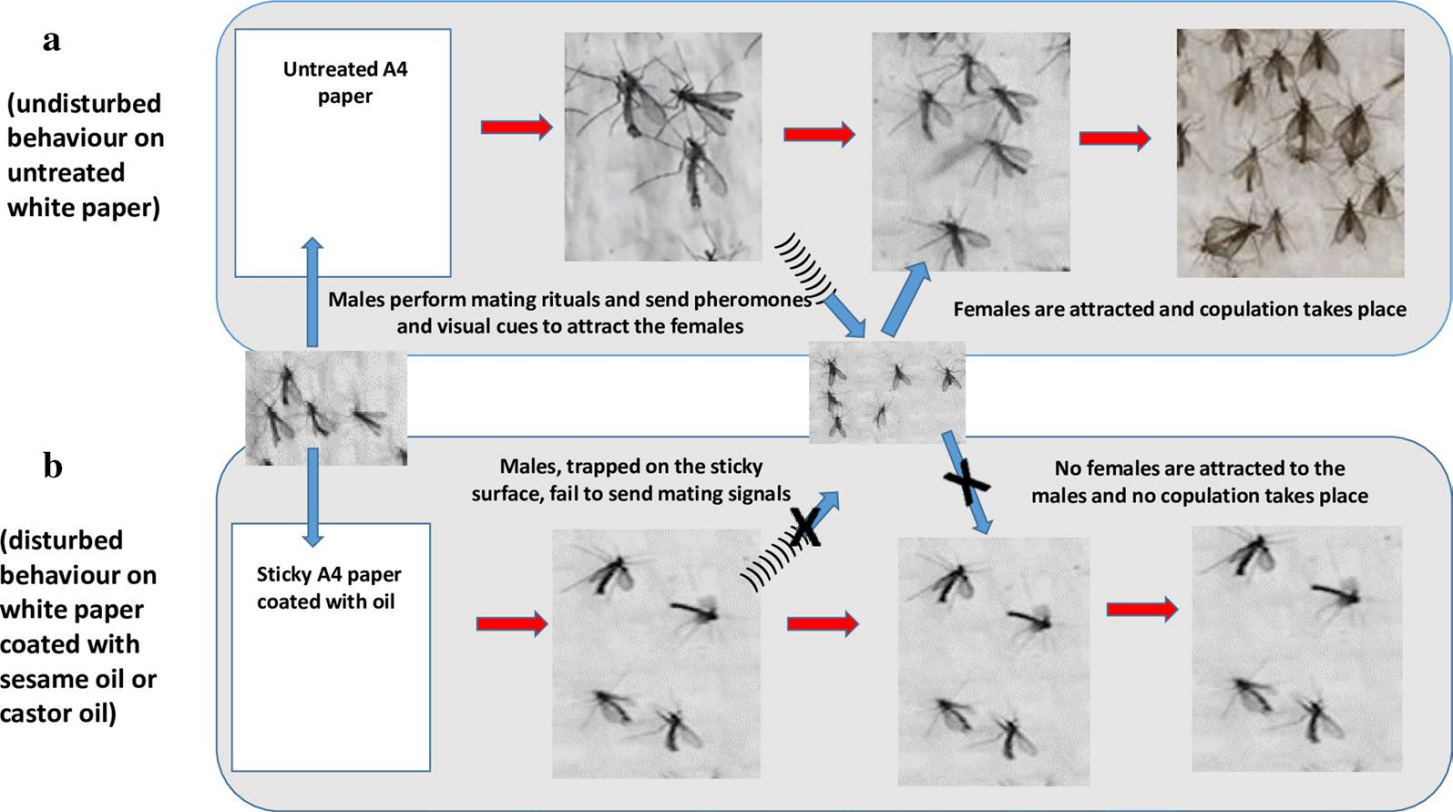
Conceptual diagram proposing how sticky traps capture *Phlebotomus orientalis* and lead to male-biased collection. Under undisturbed conditions (i.e. natural behaviour) (**a**), males of *P. orientalis* land on the bright surface of clean un-sticky white paper and perform mating rituals, releasing pheromones and playing love songs, to attract females that may successfully copulate with them. On sticky traps made of same white papers coated with oil (**b**), the males would die as they land on the paper and therefore fail to attract the females; resulting in the highly male-biased sex ratio

As observed in prior studies [6, 7, 9, 16, 17, 32], males represented most of the *P. orientalis* individuals captured on the sticky traps, in the present study. Moncaz et al. [16], addressed this phenomenon in more detail. They reported that although baiting LHT’s with CO_2_, emanating from yeast sugar extract increased the number of females 8-fold and the number of males by 0.4-fold, the number of females captured on LHT’s remained extremely low as compared to number of males [16]. The authors related this observation to the previous report of Ashford [33] who suggested that *P. orientalis* males utilize horizontal surfaces to form mating swarms. We support the view that the high attraction of these males to the sticky traps is due to mating behaviour. However, as an alternative hypothesis to the swarming explanation, we suggest that the bright surface of the sticky traps presents an arena where males can perform courtship rituals, including dance, love-songs, and pheromone communication, which attract the females for copulation (Fig. 8). However, due to their trapping by the sticky oil and the subsequent premature death, males fail to complete the courtship display and the attraction of the females. Our observations supported this hypothesis, as we routinely saw many male sand flies (most probably *P. orientalis*) landing on the clean un-sticky white papers placed horizontally on the ground. These males displayed a curious behaviour that consisted of straight-line running. Copulation readily occurred with the females that followed them in landing on the paper. This explanation does not exclude the possibility that males of *P. orientalis* may aggregate or lek near hosts as reported for a number of sand flies, including *Lutzomyia longipalpis* [34]. We must also account for the possibility of the sex specific difference in attraction to different wavelengths of light, as previously reported for *Lu. longipalpis* [27, 28]. It is suggested that future field and laboratory studies should utilize infrared video and sound recordings and chemical analysis to allow further investigations on the mating behaviour of *P. orientalis.* Similar studies on other species of sand flies revealed a number of courtship rituals, including dancing, love songs, body touch, wing flapping and release of sex pheromones that attract and/or stimulate the females to copulate [23, 25, 35–37].

We recommend that future studies utilize the experimental approach described in the present study to determine how sticky traps capture other important sand fly species and whether the observed behaviour of *P. orientalis* is common within the genus or the subgenus levels. In a previous report, Moncaz et al. [16] showed that the behaviour of *P. orientalis* contrasted sharply with behaviour of *P. papatasi*, which was not attracted to large horizontal surfaces (LHT’s) unless baited with CO_2_ derived from dry ice or fermenting yeast/sugar mixtures [16]. On the other hand, the authors reported that the large horizontal surfaces seemed to attract both sexes of *P. sergenti*, although the number of males captured on the traps was twice the number of females [16].

Future studies should also investigate an approach to overcome the disproportionate absence of females in sticky trap collection by using CO_2_ and other host odors and kairomones [16, 24, 38, 39]. Additionally, there remains an urgent need to explore the potential production and release of sex pheromones by *P. orientalis*. As have been done for *Lu. longipalpis* [23, 25, 40], these pheromones should be characterized, synthesized, and successfully applied to lure both males and females of the vector to the traps. Recently, a study showed that when co-located with insecticides, synthetic sex pheromones significantly reduced the incidence of canine visceral leishmaniasis and the abundance of *Lu. longipalpis* [41].

## Conclusions

In conclusion, the findings of this study provide a novel insight into the mechanism by which sticky traps capture *P. orientalis*, and can therefore help optimize their design for ecological studies and routine surveillance of the vector of leishmaniasis. By comparing results from different trap colours, spatial orientation and heights, the study shows that sticky traps are an attractant-based rather than interceptive sampling method of *P. orientalis.* The vector was more attracted to bright white and yellow sticky traps than to blue, green and red trap and was caught in negligible numbers on red and black traps. The colour discrimination between the traps appeared to be stronger under lunar and quarter-half lunar than on dark nights. The study also demonstrated a substantial increase in numbers of *P. orientalis* captured on sticky traps placed horizontally on the ground *versus* traps placed in an interceptive vertical position or raised on stools. Highly male biased sex ratios on sticky traps and observations of mating behaviour of the vector on un-sticky clean papers suggest that male *P. orientalis* use the bright surface of the traps as mating arena. However, the sticky surface resulted in their premature death and failure to attract the females. Further research should investigate the presence of sex pheromones in *P. orientalis* and provide further improvements of this important sand fly sampling method.

## Supplementary information

**Additional file 1: Table S1.** Pairwise Comparisons of numbers of *P. orientalis* captured on different vertical coloured sticky traps during quarter-lunar to half lunar nights in Below village, Gedarif state, Sudan (April 2016). **Table S2.** Pairwise comparisons of numbers of *P. orientalis* captured on different vertical coloured sticky traps during full lunar nights in Below village, Gedarif state, Sudan (2017). **Table S3.** Pairwise comparisons of numbers of *P. orientalis* captured on different vertical coloured sticky traps during full dark nights in Below village, Gedarif state, Sudan (2017). **Table S4.** Pairwise comparisons of numbers of *P. orientalis* captured on different horizontal coloured sticky traps during full dark nights in Below village, Gedarif state, Sudan (2018).

## Data Availability

All data used to generate the conclusions of this article are included within the article and its additional files. Raw data can be shared with other researchers upon specific request.

## Abbreviations

VL: Visceral leishmaniasis
ST: Sticky traps
IQR: Interquartile range
N: Number of replicates
