# Supplementary material for: Understanding sand fly sampling methods: sticky traps are attraction-based and not interceptive sampling tools of Phlebotomus orientalis
Source: Parasit Vectors. 2020 Jul 31;13:389. doi: 10.1186/s13071-020-04249-1 (PMC7393830; doi:10.1186/s13071-020-04249-1)
Supplement: Supplementary file 1 — Additional file 1: Table S1. Pairwise Comparisons of numbers of P. orientalis captured on different vertical coloured sticky traps during quarter-lunar to half lunar nights in Below village, Gedarif state, Sudan (April 2016). Table S2. Pairwise comparisons of numbers of P. orientalis captured on different vertical coloured sticky traps during full lunar nights in Below village, Gedarif state, Sudan (2017). Table S3. Pairwise comparisons of numbers of P. orientalis captured on different vertical coloured sticky traps during full dark nights in Below village, Gedarif state, Sudan (2017). Table S4. Pairwise comparisons of numbers of P. orientalis captured on different horizontal coloured sticky traps during full dark nights in Below village, Gedarif state, Sudan (2018). [file 13071_2020_4249_MOESM1_ESM.docx]

| **Additional file 1: Table S1. Pairwise Comparisons of numbers of *Phlebotomus orientalis* captured on different vertical coloured sticky traps during quarter-lunar to half lunar nights in Below village, Gedarif state, Sudan (April 2016).** | | | | | |  |
| --- | --- | --- | --- | --- | --- | --- |
| Trap colour comparison | Test Statistic | Std. Error | Std. Test Statistic | Sig. | Adj. Sig.^a^ | |
| Black-Red | -10.575 | 9.274 | -1.140 | .254 | 1.000 | |
| Black-Transparent | -20.236 | 9.380 | -2.157 | .031 | .465 | |
| Black-Yellow | -38.816 | 9.176 | -4.230 | .000 | .000 | |
| Black-White | -39.725 | 9.176 | -4.329 | .000 | .000 | |
| Red-Transparent | -9.661 | 9.020 | -1.071 | .284 | 1.000 | |
| Red-Yellow | -28.240 | 8.808 | -3.206 | .001 | .020 | |
| Red-White | -29.149 | 8.808 | -3.309 | .001 | .014 | |
| Transparent-Yellow | -18.580 | 8.920 | -2.083 | .037 | .559 | |
| Transparent-White | -19.489 | 8.920 | -2.185 | .029 | .433 | |
| Yellow-White | .909 | 8.705 | .104 | .917 | 1.000 | |
| Independent-Samples Kruskalis-Wallis Test: Test statistics= 29.479; N = 103; df =4 ; P<0.001  Each row tests the null hypothesis that the Sample 1 and Sample 2 distributions are the same. Asymptotic significances (2-sided tests) are displayed. The significance level is .05. | | | | | |  |
| a. Significance values have been adjusted by the Bonferroni correction for multiple tests. | | | | | |  |

| **Additional file 1: Table S2. Pairwise Comparisons of numbers of *Phlebotomus orientalis* captured on different vertical coloured sticky traps during full lunar nights in Below village, Gedarif state, Sudan (2017).** | | | | | |
| --- | --- | --- | --- | --- | --- |
| Sample 1-Sample 2 | Test Statistic | Std. Error | Std. Test Statistic | Sig. | Adj. Sig.^a^ |
| Red-Black | .598 | 7.489 | .080 | .936 | 1.000 |
| Red-Blue | -18.223 | 7.489 | -2.433 | .015 | .314 |
| Red-Yellow | -18.786 | 8.473 | -2.217 | .027 | .559 |
| Red-Green | -19.369 | 8.050 | -2.406 | .016 | .339 |
| Red-Transparent | -24.723 | 7.489 | -3.301 | .001 | .020 |
| Red-White | -31.973 | 7.489 | -4.269 | .000 | .000 |
| Black-Blue | -17.625 | 7.235 | -2.436 | .015 | .312 |
| Black-Yellow | -18.187 | 8.249 | -2.205 | .027 | .577 |
| Black-Green | -18.771 | 7.815 | -2.402 | .016 | .342 |
| Black-Transparent | -24.125 | 7.235 | -3.334 | .001 | .018 |
| Black-White | -31.375 | 7.235 | -4.336 | .000 | .000 |
| Blue-Yellow | .563 | 8.249 | .068 | .946 | 1.000 |
| Blue-Green | 1.146 | 7.815 | .147 | .883 | 1.000 |
| Blue-Transparent | 6.500 | 7.235 | .898 | .369 | 1.000 |
| Blue-White | 13.750 | 7.235 | 1.900 | .057 | 1.000 |
| Yellow-Green | -.583 | 8.762 | -.067 | .947 | 1.000 |
| Yellow-Transparent | 5.938 | 8.249 | .720 | .472 | 1.000 |
| Yellow-White | 13.188 | 8.249 | 1.599 | .110 | 1.000 |
| Green-Transparent | 5.354 | 7.815 | .685 | .493 | 1.000 |
| Green-White | 12.604 | 7.815 | 1.613 | .107 | 1.000 |
| Transparent-White | -7.250 | 7.235 | -1.002 | .316 | 1.000 |
| **Independent-Samples Kruskalis-Wallis Test: Test statistics= 30.957; N = 50; df =6; P<0.001**  Each row tests the null hypothesis that the Sample 1 and Sample 2 distributions are the same.  Asymptotic significances (2-sided tests) are displayed. The significance level is .05.  a. Significance values have been adjusted by the Bonferroni correction for multiple tests. | | | | | |
|  | | | | | |

| **Additional file 1: Table S3. Pairwise Comparisons of numbers of *P. orientalis* captured on different vertical coloured sticky traps during full dark nights in Below village, Gedarif state, Sudan (2017).** | | | | | |
| --- | --- | --- | --- | --- | --- |
| Sample 1-Sample 2 | Test Statistic | Std. Error | Std. Test Statistic | Sig. | Adj. Sig.^a^ |
| Black-Red | -3.375 | 5.798 | -.582 | .561 | 1.000 |
| Black-Blue | -7.375 | 5.798 | -1.272 | .203 | 1.000 |
| Black-Yellow | -8.750 | 5.798 | -1.509 | .131 | 1.000 |
| Black-White | -12.875 | 5.798 | -2.220 | .026 | .554 |
| Black-Green | -15.750 | 5.798 | -2.716 | .007 | .139 |
| Black-Transparent | -17.500 | 5.798 | -3.018 | .003 | .053 |
| Red-Blue | -4.000 | 5.798 | -.690 | .490 | 1.000 |
| Red-Yellow | -5.375 | 5.798 | -.927 | .354 | 1.000 |
| Red-White | -9.500 | 5.798 | -1.638 | .101 | 1.000 |
| Red-Green | -12.375 | 5.798 | -2.134 | .033 | .689 |
| Red-Transparent | -14.125 | 5.798 | -2.436 | .015 | .312 |
| Blue-Yellow | 1.375 | 5.798 | .237 | .813 | 1.000 |
| Blue-White | 5.500 | 5.798 | .949 | .343 | 1.000 |
| Blue-Green | 8.375 | 5.798 | 1.444 | .149 | 1.000 |
| Blue-Transparent | 10.125 | 5.798 | 1.746 | .081 | 1.000 |
| Yellow-White | 4.125 | 5.798 | .711 | .477 | 1.000 |
| Yellow-Green | -7.000 | 5.798 | -1.207 | .227 | 1.000 |
| Yellow-Transparent | 8.750 | 5.798 | 1.509 | .131 | 1.000 |
| White-Green | -2.875 | 5.798 | -.496 | .620 | 1.000 |
| White-Transparent | 4.625 | 5.798 | .798 | .425 | 1.000 |
| Green-Transparent | 1.750 | 5.798 | .302 | .763 | 1.000 |
| **Independent-Samples Kruskalis-Wallis Test: Test statistics= 14.705; N =28; df =6 ; P=0.023**  Each row tests the null hypothesis that the Sample 1 and Sample 2 distributions are the same.  Asymptotic significances (2-sided tests) are displayed. The significance level is .05. | | | | | |
| a. Significance values have been adjusted by the Bonferroni correction for multiple tests. | | | | | |

| **Additional file 1: Table S4. Pairwise Comparisons of numbers of *Phlebotomus orientalis* captured on different horizontal coloured sticky traps during full dark nights in Below village, Gedarif state, Sudan (2018).** | | | | | |
| --- | --- | --- | --- | --- | --- |
| Sample 1-Sample 2 | Test Statistic | Std. Error | Std. Test Statistic | Sig. | Adj. Sig.^a^ |
| Green-Red | 6.688 | 6.883 | .972 | .331 | 1.000 |
| Green-Blue | -7.875 | 6.883 | -1.144 | .253 | 1.000 |
| Green-Transparent | 16.125 | 6.883 | 2.343 | .019 | .287 |
| Green-Yellow | 26.063 | 6.883 | 3.787 | .000 | .002 |
| Green-White | 34.000 | 6.883 | 4.940 | .000 | .000 |
| Red-Blue | -1.187 | 6.883 | -.173 | .863 | 1.000 |
| Red-Transparent | -9.437 | 6.883 | -1.371 | .170 | 1.000 |
| Red-Yellow | -19.375 | 6.883 | -2.815 | .005 | .073 |
| Red-White | -27.312 | 6.883 | -3.968 | .000 | .001 |
| Blue-Transparent | 8.250 | 6.883 | 1.199 | .231 | 1.000 |
| Blue-Yellow | 18.188 | 6.883 | 2.642 | .008 | .123 |
| Blue-White | 26.125 | 6.883 | 3.796 | .000 | .002 |
| Transparent-Yellow | -9.937 | 6.883 | -1.444 | .149 | 1.000 |
| Transparent-White | -17.875 | 6.883 | -2.597 | .009 | .141 |
| Yellow-White | 7.938 | 6.883 | 1.153 | .249 | 1.000 |
| **Independent-Samples Kruskalis-Wallis Test: Test statistics= 35.017; N =48; df =5 ; P<0.001**  Each row tests the null hypothesis that the Sample 1 and Sample 2 distributions are the same.  Asymptotic significances (2-sided tests) are displayed. The significance level is .05. | | | | | |
| a. Significance values have been adjusted by the Bonferroni correction for multiple tests. | | | | | |
